# A Dual Role for FADD in Human Precursor T-Cell Neoplasms

**DOI:** 10.3390/ijms232315157

**Published:** 2022-12-02

**Authors:** José Luis Marín-Rubio, Laura Vela-Martín, Jack Gudgeon, Eduardo Pérez-Gómez, Frances R. Sidgwick, Matthias Trost, Debbie L. Cunningham, Javier Santos, José Fernández-Piqueras, María Villa-Morales

**Affiliations:** 1 Laboratory for Biological Mass Spectrometry, Biosciences Institute, Newcastle University, Newcastle-upon-Tyne NE2 4HH, UK; 2 Centro de Biología Molecular Severo Ochoa (CBMSO), Consejo Superior de Investigaciones Científicas-Universidad Autónoma de Madrid (CSIC-UAM), 28049 Madrid, Spain; 3 Departamento de Biología, Universidad Autónoma de Madrid, 28049 Madrid, Spain; 4 Area of Genetics and Genomics, IIS Fundación Jiménez Díaz, 28040 Madrid, Spain; 5 Departamento de Bioquímica y Biología Molecular, Universidad Complutense de Madrid, 28005 Madrid, Spain; 6 Instituto de Investigación Hospital 12 de Octubre, 28041 Madrid, Spain; 7 School of Biosciences, University of Birmingham, Edgbaston, Birmingham B15 2TT, UK; 8 Instituto Universitario de Biología Molecular (IUBM), UAM, 28049 Madrid, Spain

**Keywords:** precursor T-cell neoplasms, T-cell acute lymphoblastic leukemia/T-cell lymphoblastic lymphoma (T-ALL/LBL), FADD, transcriptomics, interactomics, canonical and non-canonical functions

## Abstract

A reduction in *FADD* levels has been reported in precursor T-cell neoplasms and other tumor types. Such reduction would impact on the ability of tumor cells to undergo apoptosis and has been associated with poor clinical outcomes. However, FADD is also known to participate in non-apoptotic functions, but these mechanisms are not well-understood. Linking FADD expression to the severity of precursor T-cell neoplasms could indicate its use as a prognostic marker and may open new avenues for targeted therapeutic strategies. Using transcriptomic and clinical data from patients with precursor T-cell neoplasms, complemented by in vitro analysis of cellular functions and by high-throughput interactomics, our results allow us to propose a dual role for FADD in precursor T-cell neoplasms, whereby resisting cell death and chemotherapy would be a canonical consequence of FADD deficiency in these tumors, whereas deregulation of the cellular metabolism would be a relevant non-canonical function in patients expressing FADD. These results reveal that evaluation of FADD expression in precursor T-cell neoplasms may aid in the understanding of the biological processes that are affected in the tumor cells. The altered biological processes can be of different natures depending on the availability of FADD influencing its ability to exert its canonical or non-canonical functions. Accordingly, specific therapeutic interventions would be needed in each case.

## 1. Introduction

Precursor T-cell neoplasms (T-ALL/LBL, T-cell acute lymphoblastic leukemia/T-cell lymphoblastic lymphoma) are a type of aggressive hematological cancer derived from immature T-cells in various stages of differentiation, mostly affecting children and adolescent males [1]. A significant reduction in Fas-associated proteins with death domain (FADD) levels has been reported in precursor T-cell neoplasms by our group and others [2,3,4]. Moreover, a decrease in FADD has been associated with poor clinical outcomes, such as drug resistance, inferior survival, increased recurrence or metastasis in many tumor types, such as human non-small cell lung cancer [5], thyroid adenoma/adenocarcinoma [6] or acute myeloid leukemia [7]. 

However, the role of FADD in cancer is controversial, but apparent discrepancies between studies could be explained by the fact that FADD exhibits both apoptotic and non-apoptotic roles. Therefore, this activity could be dependent on cell type and would determine the outcome of each tumor [5,6,8,9,10,11]. Unfortunately, the mechanisms regulating its participation in non-apoptotic functions are not well-understood. Linking FADD expression to the severity of precursor T-cell neoplasms could indicate its use as a prognostic marker and may open new avenues for targeted therapeutic strategies. Furthermore, whether FADD expression has implications beyond the impairment of FADD-dependent programmed cell death is yet to be elucidated in human precursor T-cell neoplasms, thus representing a pertinent topic for cancer research. 

In this study, we set out to gain insight into the clinical implications of FADD expression, and to explore the landscape of dysregulated cell signaling events in human precursor T-cell neoplasms, taking FADD expression as the defining variable. For such purpose, we analyzed disease-free survival of patients with a precursor T-cell neoplasm exhibiting differential *FADD* levels, followed by analysis of transcriptomic and clinical data from a cohort of 264 patients. To add further biological meaning to gene expression data, we performed mass-spectrometry-based quantitative proteomics to validate our results at the protein level, using state-of-the-art data-independent acquisition mass spectrometry (DIA-MS). Protein–protein interactions (PPIs) are one of the major mechanisms for controlling protein functions in various cellular processes [12,13,14]. Furthermore, dysfunction of PPIs is one of the major causes of many diseases, including cancer [15]. Global mapping of PPIs has become a major goal in mass spectrometry [16]. Identification of PPIs at a global level will provide important insights into the regulation of cellular processes [12]. A combinatorial approach increases the number of candidate proteins in comparison to single analyses [17]. Herein, we applied a DIA-MS-based proteomic method to screen specific protein interactions of endogenous FADD proteins in precursor T-cell neoplasm-derived JURKAT cell lines. Thus, this is the first analysis of endogenous FADD using a DIA-MS-based quantitative proteomic approach in non-apoptotic conditions [18,19,20,21,22,23,24,25].

In this work, we explore the clinical significance of FADD expression in precursor T-cell neoplasms and the enrichment of distinctive genetic signatures in patients, according to *FADD* levels. We reveal that reduced *FADD* levels are associated with poor prognosis in precursor T-cell neoplasms, and that such reductions determined a significant enrichment of oncogenic gene signatures, including resistance to chemotherapy. However, those tumors not exhibiting reduced *FADD* levels showed significant enrichment of multiple gene signatures of energy metabolism. This is supported by co-Immunoprecipitation-Mass Spectrometry (coIP-MS) revealing significant interactions of FADD with proteins related with energy metabolism. We propose a dual role for FADD in precursor T-cell neoplasms, where FADD reduction would affect its canonical function, programmed cell death, leading to cell death resistance and subsequent chemotherapy resistance. FADD preservation in tumor cells, however, would affect its non-canonical functions, and our results indicate that changes in energy metabolism are such a relevant non-canonical function in precursor T-cell neoplasms.

## 2. Results

### 2.1. Reduced FADD Levels Associate with Poor Prognosis in Precursor T-Cell Neoplasms

**Figure 1:**
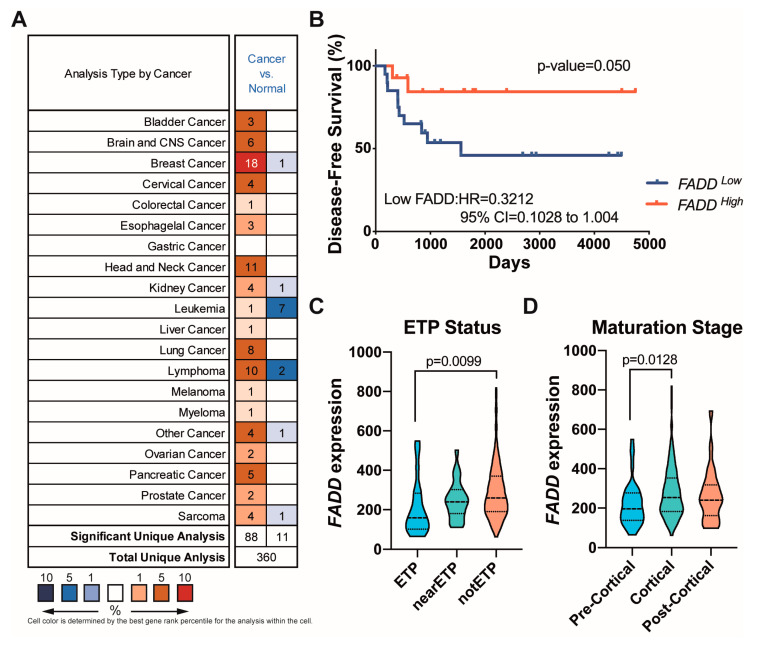
Reduced FADD levels associated with poor prognosis in precursor T-cell neoplasms. (**A**) Analysis of FADD expression in cancer types. The gene summary for FADD in cancers was gained from Oncomine^TM^ Research Edition (http://www.oncomine.org (accessed on 18 September 2018)). (**B**) Kaplan–Meier disease-free survival curve analysis in precursor T-cell neoplasms with high and low *FADD* expression obtained from the dataset published in the Ref. [29]. HR, hazard ratio (Mantel–Haenszel); 95% CI, 95% confidence interval of ratio. (**C**,**D**) Violin plots representing *FADD* expression (DESeq2 normalized counts) in patients belonging to the ETP, nearETP, and notETP subtypes and in patients belonging to the Pre-Cortical, Cortical, and Post-Cortical subtypes (as defined in the Ref. [26]), respectively. Krustal–Wallis was used to test for statistical significance.

*FADD* downregulation is more frequent in leukemia and lymphoma than in any other cancer type (Figure 1A). We performed a disease-free survival analysis from 34 patients with a precursor T-cell neoplasm, which revealed significantly shortened disease-free survival for patients exhibiting low *FADD* expression (Figure 1B). Similarly, in 57 patients with acute myeloid leukemia (AML), we observed that *FADD* expression was significantly lower in the group of patients that did not survive after 3 years (Supplementary Figure S2). To further investigate the implication of FADD expression in the outcome of precursor T-cell neoplasms, we performed additional analyses of the clinic-pathological data associated to 264 patients (TARGET cohort), considering *FADD* levels. A significant variation (*p*-value = 0.0103) was found by multiple comparison of *FADD* expression in patients belonging to three different subtypes: Early T-cell Precursor (ETP), near-ETP, and not-ETP (defined in [26]). Our results revealed that patients belonging to the ETP clinical subtype exhibited significantly lower *FADD* expression levels than those defined as not-ETP (adjusted *p*-value = 0.0099) (Figure 1C). Additionally, a multiple comparison of *FADD* expression revealed a significant variation (*p*-value = 0.0164) in patients classified according to their maturation stage [26] as Pre-Cortical, Cortical, and Post-Cortical subtypes. In particular, we observed that *FADD* expression was significantly lower in patients belonging to the subtype defined as Pre-Cortical, in comparison with those defined as Cortical (adjusted *p*-value = 0.0128) (Figure 1D). Since the ETP subtype is considered a very high-risk group [27] and the Cortical subtype has shown the best clinical outcomes [28], these results indicate that *FADD* expression may have a prognostic value for precursor T-cell neoplasms.

### 2.2. Patients with Precursor T-Cell Neoplasms Exhibiting Reduced FADD Levels Showed a Significant Association with Oncogenic Signatures

**Figure 2:**
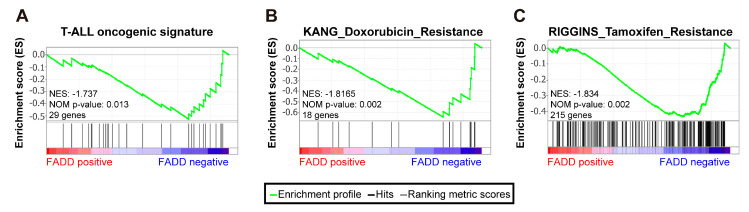
The *FADD-negative* phenotype showed a significant association with oncogenic signatures. Gene Set Enrichment Analysis was performed in 264 patients with precursor T-cell neoplasm, based on their *FADD* expression levels, which defined *FADD-negative* and *FADD-positive* phenotypes. These signatures were significantly enriched in the *FADD-negative* phenotype. (**A**) This signature was created ad hoc based on previous literature and is defined in Supplementary Table S1. (**B**,**C**) These signatures were selected from the Molecular Signatures Database, and their systematic names are M3323 (Doxorubicin Resistance) and M15835 (Tamoxifen Resistance). The green curve corresponds to the enrichment score (ES) curve, which is the running sum of the weighted enrichment score obtained with the GSEA software. NES, normalized enrichment score; *p*, nominal *p* value.

We sought to explore the relationship between *FADD* expression and the landscape of deregulated cell signaling events in human precursor T-cell neoplasms. To do so, we performed bioinformatics analyses on whole transcriptional profiles of the 264 patients with precursor T-cell neoplasms (TARGET cohort), which revealed that patients exhibited different genetic signatures depending on *FADD* expression. In particular, tumors exhibiting reduced *FADD* levels (named as the *FADD-negative* group on the basis of a continuous phenotype label) showed significant enrichment of a gene signature gathering the most relevant genes in T-cell acute lymphoblastic leukemia (T-ALL), defined as the T-ALL_oncogenic signature and created ad hoc based on previous literature [30] (Figure 2A). This suggests that resisting programmed cell death, which is the canonical function of FADD, could represent a major hallmark of cancer in these patients with reduced *FADD* levels. One consequence of programmed cell death impairment in tumor cells is the development of resistance to anti-cancer therapy [31]. In support of this notion, we also observed a significant association with gene signatures related to chemotherapy resistance in precursor T-cell neoplasms exhibiting reduced *FADD* levels (Figure 2B,C).

### 2.3. Precursor T-Cell Tumor Cells Lacking FADD Showed Reduced Sensitivity to Chemotherapeutic Agents

**Figure 3:**
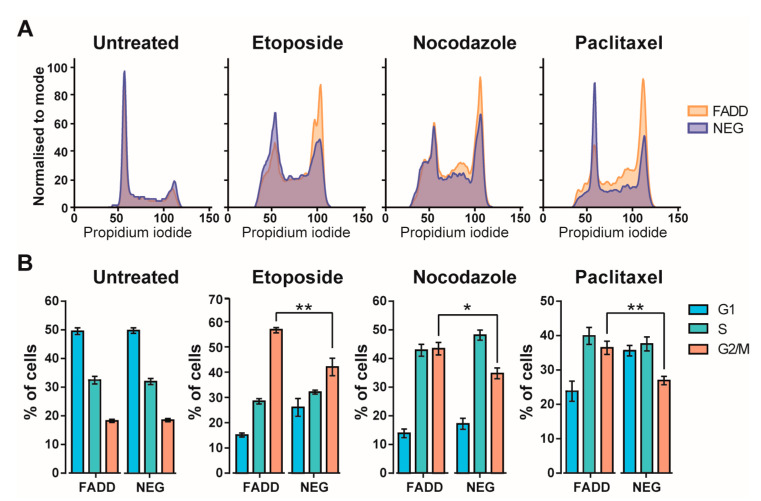
Pharmacological arrest at the G2/M stage of the cell cycle. The cell cycle of the FADD-expressing (FADD) and FADD-deficient (NEG) JURKAT cell lines was evaluated without any treatment (untreated) or after treatment with 100 nM etoposide, 50 ng/mL nocodazole, and 10 nM paclitaxel for 18 h. (**A**) Representative histograms of cells stained with propidium iodine showing DNA content distribution. Cell cycle phase distribution was analyzed by flow cytometry. (**B**) Bar chart of cell cycle distribution from six independent experiments. One-way ANOVA was used to test for statistical significance: * *p* ≤ 0.05; ** *p* ≤ 0.01. Error bars represent the standard error of the mean (SEM).

We found a significant association between *FADD* reduction and chemotherapy resistance signatures. To validate this result, we sought to investigate whether FADD levels would affect the response of precursor T-cell tumor cells to pharmacological cell cycle arrest with several chemotherapeutic agents. We performed in vitro analysis of cell cycle phase distribution in FADD-expressing (JURKAT-FADD) and FADD-deficient (JURKAT-NEG) precursor T-cell neoplasm-derived JURKAT T-cells, either in untreated cells or upon specific pharmacological treatments (Figure 3). Untreated cell lines showed a similar distribution of cell cycle phases. Upon G2/M-arrest with etoposide, nocodazole, or paclitaxel, the extent of the arrest was significantly weaker in FADD-deficient cells. This indicates that precursor T-cell tumor cells with reduced FADD levels are more resistant to the pharmacological arrest at G2/M executed by some chemotherapeutic agents. This may explain why precursor T-cell neoplasm patients with reduced *FADD* levels display genetic signatures of resistance to anti-cancer therapies.

### 2.4. Energy Metabolism Signatures Are Significantly Enriched in Patients Not Exhibiting FADD Reduction

**Figure 4:**
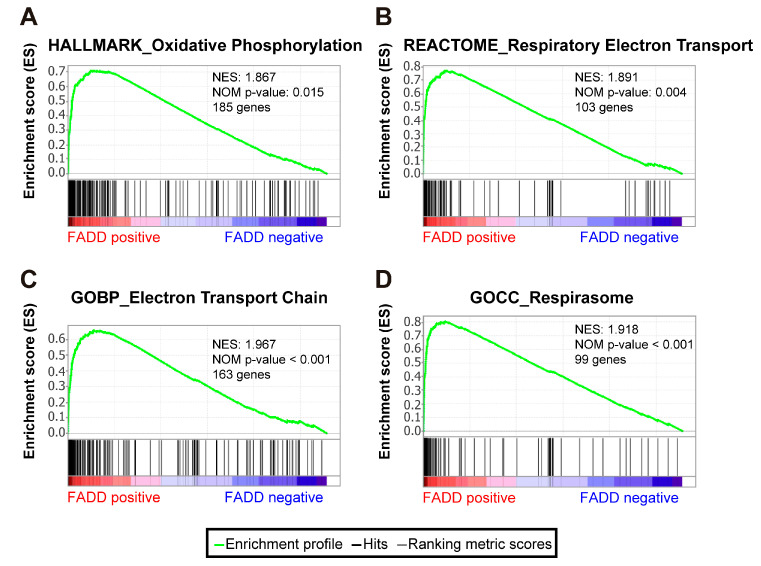
The *FADD*-positive phenotype showed a significant association with energy metabolism signatures. Gene Set Enrichment Analysis was performed for 264 patients with precursor T-cell neoplasm, based on their *FADD* expression levels, which defined *FADD*-negative and *FADD*-positive phenotypes. These signatures were significantly enriched in the *FADD*-positive phenotype and selected from the Molecular Signatures Database. Their systematic names are M5936 (Hallmark_Oxidative Phosphorylation) (**A**), M893 (Reactome_Respiratory Electron Transport) (**B**), M13293 (GOBP_Electron Transport Chain) (**C**) and M25889 (GOCC_Respirasome) (**D**). The green curve corresponds to the enrichment score (ES) curve, which is the running sum of the weighted enrichment score obtained with the GSEA software. NES, normalized enrichment score; *p*, nominal *p*-value.

While low *FADD* expression levels were associated with poor prognosis and chemotherapy resistance, there is a subgroup of precursor T-cell tumors with moderate/high *FADD* expression levels. We analyzed those patients (*FADD*-positive group) following the same approach described previously. As expected, considering the canonical function of FADD, genetic signatures related to programmed cell death were significantly enriched in the *FADD*-positive group (Supplementary Figure S3). Besides this, a striking association with a non-canonical function was also observed, where multiple gene signatures of energy metabolism were significantly enriched (Figure 4). This suggests that in those precursor T-cell tumors that are able to execute FADD-dependent programmed cell death, FADD may have a role in a different hallmark of cancer, deregulation of the cellular metabolism.

### 2.5. Interactome Analysis Using DIA-MS Confirms FADD Participation in Energy Metabolism Processes

**Figure 5:**
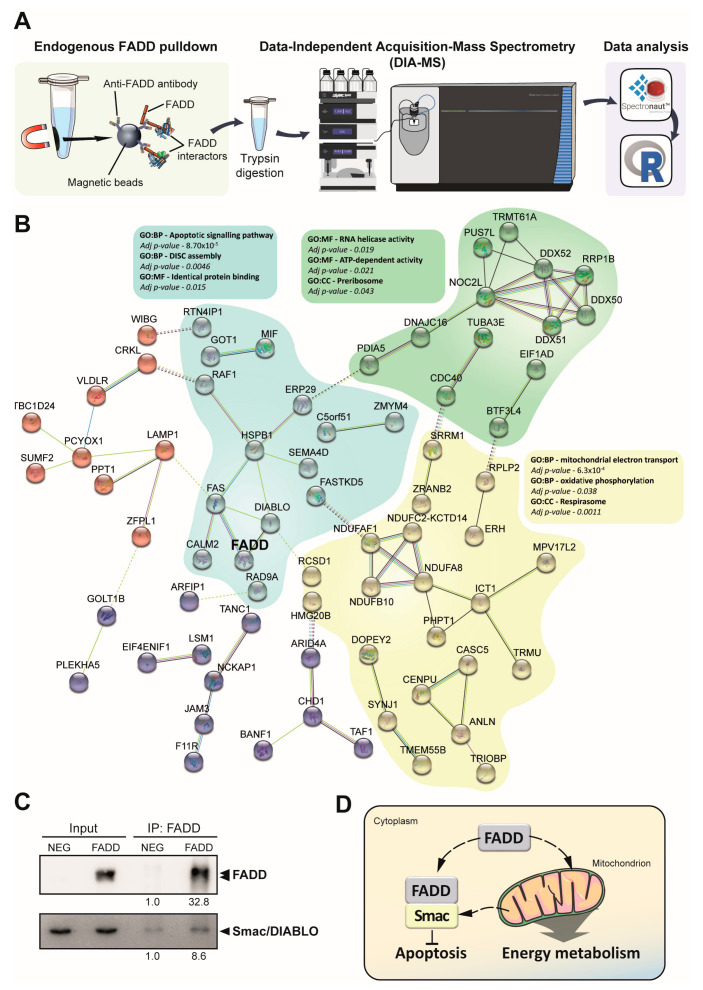
Interactome analysis using DIA-MS confirms FADD participation in energy metabolism processes. (**A**) Workflow of co-Immunoprecipitation-Mass Spectrometry. Endogenous FADD protein was co-immunoprecipitated from FADD-expressing (FADD) and FADD-deficient (NEG) JURKAT cell lines using magnetic beads. Samples were trypsin-digested and injected into the mass spectrometer for data-independence acquisition. Finally, the raw data were analyzed with Spectronaut software and R. (**B**) STRING interaction map of significant proteins that interact with FADD in JURKAT cells compared to JURKAT-deficient FADD (FADD NEG). Proteins were clustered based on K-means into five clusters. The functional enrichment analysis and adjusted *p*-value (adj. *p*-value) are shown for the three main clusters. (**C**) Validation of DIABLO–FADD interaction. The input and immunoprecipitated (IP) fractions were separated by SDS-PAGE and blotted with antibodies against anti-FADD and -DIABLO. The densitometry values are shown below. (**D**) Schematic representation to show interactions of FADD with proteins involved in different biological processes. Under non-apoptotic conditions, FADD interacts with DIABLO/Smac, which hampers FADD function inducing apoptosis. FADD also interacts with several proteins involved in the energy metabolism.

To confirm the participation of FADD in energy metabolism, we studied the interactome of endogenous FADD in tumor T-cells. Co-immunoprecipitation-Mass Spectrometry (coIP-MS) was performed using data-independent acquisition (DIA) (Figure 5A) to analyze the specific protein–protein interaction networks of FADD. We identified 117 proteins across five biological replicates that significantly interact with FADD (Supplementary Table S2). We performed bioinformatics analysis of these 117 putative interactors of FADD, which provided an overview of the functions involved. These proteins were evaluated for functional enrichment analysis and the results revealed that various biological processes were involved. As expected, 25 out of 117 proteins (21.4%) belonged to a cluster with biological processes related to FADD-dependent apoptosis, such as the apoptotic signaling pathway or DISC assembly (Figure 5B). Notably, 29 out of these 117 proteins (24.8%) belonged to a cluster with biological processes related to the energy metabolism, such as mitochondrial electron transport, oxidative phosphorylation, or respirasome (Figure 5B), reinforcing our previous results of enriched genetic signatures. A combinatorial approach increases the number of candidate proteins in comparison to single analyses, but requires additional validation, such as immunoblot analysis [17]. For verification of mass spectrometry results, endogenous FADD interaction with Smac/DIABLO (Diablo IAP-Binding mitochondrial protein) was confirmed by immunoprecipitation (Figure 5C).

Taken together, these results indicate that FADD interaction with certain proteins such as Smac/DIABLO regulates apoptosis (Figure 5D). However, we demonstrated a significant enrichment in protein–protein interactions between FADD and several proteins with a role in energy metabolism processes. This non-canonical function would be particularly relevant in precursor T-cell neoplasms where FADD is available.

## 3. Discussion

A major problem for precursor T-cell neoplasms is the lack of reliable prognostic factors besides minimal residual disease [1,28]. Identifying markers with a prognostic value is necessary to improve the clinical management of these patients, particularly of those who are refractory to treatment and those who relapse. In this study, our results revealed that disease-free survival was significantly shortened for T-ALL patients exhibiting low *FADD* expression. In line with this, we observed that *FADD* levels were significantly lower in AML patients surviving less than 3 years after diagnosis. The analysis of clinic-pathological parameters in the much larger TARGET cohort, consisting of 264 patients with a precursor T-cell neoplasm, revealed additional correlations between *FADD* expression and poor outcome factors. Early T-cell Precursor (ETP) and Pre-Cortical patients displayed lower *FADD* levels than the not-ETP and Cortical, respectively. The ETP subtype is an independent entity within the T-lymphoblastic leukemia/lymphoma updated classification [32]. Despite no clinical relevance being definitively assigned to subtypes of precursor T-cell neoplasms [32], accumulating evidence indicates that the ETP subtype is a group with a worse prognosis [27,33]. Regarding the maturation stage of the tumors, the best outcomes have been observed in the cortical subtype [28].

These results are in line with previous evidence indicating that FADD expression might be correlated to patient prognosis. In a study in AML [7], 100% of patients with long-term complete remission showed high FADD levels in leukemic cells, while 82% of those patients exhibiting chemotherapy resistance or relapse showed reduced FADD levels. Thus, the absence or reduction in FADD expression in leukemic cells at diagnosis would be indicative of a poor prognosis regarding complete remission, event-free survival, and overall survival. More recent studies by our group reported that FADD reduction might be a frequent mechanism whereby Fas-mediated apoptotic signaling would be affected in precursor T-cell neoplasms [2,4,34]. FADD reduction has also been observed in a variety of tumor types, such as non-small-cell lung cancer [5], hepatocellular carcinoma [35], or thyroid cancer [6], indicating that FADD expression can be lost in mouse and human cancer cells and could be used as a prognostic factor for an insufficient response to chemotherapy [36].

In silico analysis of whole transcriptomics data from the cohort of 264 patients with precursor T-cell neoplasm has allowed us to identify specific genetic signatures in patients with reduced *FADD* levels (*FADD-negative* phenotype). These tumors showed significant enrichment of a gene signature consisting of the most relevant genes in T-ALL [30], suggesting that loss of FADD canonical role in apoptosis would have been instrumental in the pathogenesis of these tumors. Furthermore, those tumors also exhibited significant enrichment of gene signatures related to chemotherapy resistance, which is in agreement with previous reports indicating that the development of resistance to anti-cancer therapy is a consequence of resisting cell death in cancer [31,37], and particularly in precursor T-cell neoplasm cells [38]. The arrest at particular stages or transitions in the tumor cell cycle is one biological consequence of anti-cancer chemotherapy [39], and a lack of sensitivity to anti-cancer therapy frequently relies on a diminished ability of the chemotherapeutic agent to induce cell cycle arrest on the tumor cell [40,41,42,43]. We have corroborated this notion by analyzing the effect of several chemotherapeutic agents on cell cycle arrest, comparing FADD-expressing and FADD-deficient JURKAT T-cells. Our results demonstrate that FADD-deficient JURKAT T-cells are more resistant to the pharmacological arrest executed by three different chemotherapeutic agents.

Altogether, this highlights the notion that resisting programmed cell death, which is a hallmark of cancer [44], would be a canonical consequence of FADD deficiency in these tumors. However, increasing evidence indicates that FADD is also relevant for non-apoptotic functions in T-cells [2,34,36], meaning FADD expression in precursor T-cell neoplasms may involve further functional implications. Non-canonical functions may be relevant in patients not exhibiting FADD reduction. In this setting, those tumors in the TARGET cohort not exhibiting reduced *FADD* levels showed a striking enrichment of multiple gene signatures related to energy metabolism. To support this observation at the molecular level, we set out to generate a scheme of the proteins that associate with FADD in non-apoptotic conditions. The molecular events underlying FADD-dependent cell death have been described in detail in recent years [20,45,46,47,48]; however, relatively little has been published about the non-apoptotic signals [18,49,50]. Importantly, this study provides the first characterization of endogenous FADD interactome in non-apoptotic conditions. Ectopic overexpression of wild type or tag-fused proteins would provide higher fold-changes and more interactions in proteomic approaches. However, FADD overexpression has been reported to produce death filaments and to induce apoptosis [51,52,53,54]. Fusion proteins can also alter interactions with other proteins [55]. Thus, our approach more accurately recapitulates the scenario of a precursor T-cell tumor cell. For the first time, endogenous FADD immunoprecipitation has been carried out in this setting, without either overexpression of FADD or cell death induction as previously done [18,19,20,22,23,24,25,56]. In this study, we used a data-independent acquisition mass spectrometry [57] strategy to identify novel proteins that interact with endogenous FADD in human T-ALL JURKAT cells. The application of this method increases the analytical depths of proteomic analysis and increases the number of potentially biologically relevant proteins [58]. Among them, 40 of 117 proteins (37.6%) were related to cellular metabolism. These constitute significant candidates with interesting potential new roles in regulation of energy metabolism as a non-canonical function of FADD. Furthermore, this implies that FADD may participate in pathogenic changes of energy metabolism in those precursor T-cell neoplasms that do not exhibit a phenotype of cell death resistance, and consequently a phenotype of resistance to chemotherapy due to FADD reduction. Changes in metabolism are frequently observed in cancer cells [59,60]. This metabolic reprogramming, known as aerobic glycolysis or the Warburg effect, allows tumor cells to sustain their fast proliferation.

Altogether, our results point towards a dual role for FADD in precursor T-cell neoplasms, where FADD reduction would affect its canonical function, programmed cell death, leading to cell death resistance and subsequent chemotherapy resistance. FADD preservation in tumor cells, however, would affect its non-canonical roles, and our results indicate that changes in energy metabolism is a relevant non-canonical function in precursor T-cell neoplasms.

In summary, these results reveal that evaluation of FADD expression in precursor T-cell neoplasms may aid in the understanding of the biological processes that are affected in the tumor cells. The biological processes altered can be of different nature depending on the availability of FADD influencing its ability to exert its canonical or non-canonical functions. Accordingly, specific therapeutic interventions would be needed in each case. Further studies of FADD interactions in patients may shed light on the molecular mechanisms whereby FADD can deregulate cellular signals in the tumor.

## 4. Materials and Methods

### 4.1. In Silico Analysis of Published Microarray Datasets

Human *FADD* mRNA expressions in T-ALL and AML were obtained from microarray datasets published in the Refs. [29,61], respectively. To carry out the comparative analysis between normal and tumor cells, we used the “differential analysis—cancer vs. normal” tool available at the Oncomine database (www.oncomine.org (accessed on 18 September 2018)).

### 4.2. RNA Sequencing and Clinical Data Analysis

Whole transcriptomic (RNA-seq) and clinical data from 264 patients of the precursor T-cell neoplasm (TARGET cohort) were available for in silico analysis. This work is, in part, based upon data generated by the Therapeutically Applicable Research to Generate Effective Treatments (TARGET) initiative, accession number phs000218 and substudy specific accession number phs000464.v19.p8 (TARGET Acute Lymphoblastic Leukemia (ALL) Expansion Phase 2), managed by the National Cancer Institute (NCI). Data for this analysis are accessible through the genotypes and phenotypes database (dbGaP, https://www.ncbi.nlm.nih.gov/projects/gap/cgi-bin/study (accessed on 2 February 2022)). Information about TARGET can be found at http://ocg.cancer.gov/programs/target (accessed on 2 February 2022). The use of data for this cohort requires dbGaP Authorized Access. Our group is an approved requester from 27 November 2020, thus omics and associated clinical data are fully available.

### 4.3. Gene Set Enrichment Analysis (GSEA)

Gene set enrichment analysis was performed using the GSEA desktop application from the Broad Institute and following instructions in the user guide [62,63]. A continuous phenotype label was created based on *FADD* expression across the TARGET cohort, and defined the *FADD*-negative and *FADD*-positive groups for comparison. Analysis was run using default parameters (1000 permutations, phenotype permutation and a False Discovery Rate (FDR) cut-off of 25%); a Pearson metric for ranking genes was also used, as recommended for continuous phenotype labels. The normalized enrichment score (NES) and nominal *p*-value were measured. The T-ALL_Oncogenic_Signature was created ad hoc based on previous literature [30], and is defined in Supplementary Table S1. The rest of the gene sets were selected from the Molecular Signatures Database (MSigDB), and their systematic names are as follows: M3323 (Doxorubicin-Resistance); M15835 (Tamoxifen-Resistance), M27436 (Reactome_Programmed Cell Death), M15303 (Reactome_Apoptosis), M5936 (Hallmark_Oxidative Phosphorylation), M893 (Reactome_Respiratory Electron Transport), M13293 (GOBP_ Electron Transport Chain) and M25889 (GOCC_Respirasome).

### 4.4. Cell Culture

JURKAT cell lines were generated from FADD-deficient JURKAT clone I2.1 (ATCC, CRL-2572; Manassas, VA, USA) by transduction with lentiviral particles carrying EX-V0108-Lv-225 (*FADD* cDNA) or its empty control EX-NEG-Lv225 (GeneCopoeia, Rockville, MD), to generate FADD-expressing (JURKAT-FADD) and FADD-deficient (JURKAT-NEG) cell lines as described before [34]. Cells were grown in RPMI (Thermo Fisher Scientific, Loughborough, UK) supplemented with 15% FBS, 1 mM sodium pyruvate, 2 mM L-glutamine, and 3 µg/mL puromycin. Cultures were maintained at 37 °C in a 5% CO2 humidified atmosphere. Additionally, we corroborated that FADD expression of stable cell lines was equivalent to the FADD endogenous level of the parental clone JURKAT A3 (A3; ATCC, CRL-2570) (Supplementary Figure S1). ATCC routinely performs cell line authentication, using STR profiling as a procedure. Cell experimentation was always performed within a period not exceeding 6 months after resuscitation and in mycoplasma-free culture conditions.

### 4.5. Cell Cycle Analysis

Cell cycle profiling using PI/RNase Staining Buffer (BD Biosciences, San Jose, CA, USA) was performed according to the manufacturer’s instructions. Phase distribution of the cell cycle was examined after 18 h with 100 nM etoposide, 5 mM hydroxyurea, 50 ng/mL nocodazole, 10 nM paclitaxel, or a double 2 mM thymidine block for 18 h and released for 6 h. Etoposide, hydroxyurea, nocodazole, paclitaxel, and thymidine were purchased from Sigma-Aldrich (Sigma-Aldrich, St. Louis, MO, USA). All analytic cytometry procedures were performed on a FACS Canto II flow cytometer (Becton–Dickinson, Franklin Lakes, NJ, USA). The Watson pragmatic fitting algorithm was used to determine cell cycle phase statistics using FlowJo v10.

### 4.6. Preparation of Cellular Extracts and Immunoprecipitation of Endogenous FADD

Whole cells were lysed with lysis buffer (50 mM Tris-HCl, pH 8.0, 1% Triton X-100, 150 mM NaCl, 1 mM EDTA) supplemented with PhosStop (Roche Diagnostics GmbH, Mannheim, Germany) and cOmplete™ Mini Protease Inhibitor Cocktail (Roche Diagnostics GmbH) for 15 min on ice. Lysed cells were passed through a 21-gauge needle to shear the DNA, centrifuged at 15,000× *g* for 15 min at 4 °C, and the supernatant was collected. Protein concentrations were determined using the BCA assay (Thermo Fisher Scientific) as per the manufacturer’s instructions. For endogenous FADD immunoprecipitation, 1 mg of protein extracts from each cell line were precleared by incubation on dynabead protein G (Thermo Fisher Scientific) alone for 30 min at 4 °C. Sixty micrograms of monoclonal anti-FADD antibody (FADD (A66-2): 556402; BD Biosciences) were covalently coupled to 1.8 mg of dynabead protein G (Thermo Fisher Scientific). The beads were incubated with antibody for 2 h at 4 °C and then washed twice with 10 volumes of 0.1 M sodium borate, pH 9.3. Next, the beads were incubated twice with 10 volumes of borate buffer containing 20 mM dimethylpimelimidate (DMP; Sigma-Aldrich) for 30 min at room temperature. The beads were washed four times with 10 volumes of ice-cold 50 mM glycine (pH 2.5) to remove unbound antibodies, then neutralized for 2 h with 0.2 M Tris-HCl (pH 8.0). This complex was incubated with pre-clearing proteins overnight at 4 °C and then washed five times with citrate phosphate buffer (pH 5.0) for use. Beads were then mixed and washed a further three times and eluted using 5% SDS in 50 mM TEAB.

### 4.7. Western Blot

Proteins were electrophoresed in Mini-PROTEAN-TGXTM Precast Gels (Bio-Rad Laboratories, Hercules, CA, USA) and then transferred to mini-sized PVDF membranes by the Tranfer Blot^®^ Turbo^TM^ Transfer System (Bio-Rad Laboratories). The peroxidase activity was developed using the WesternBright ECL Detection System (Advansta, Menlo Park, CA, USA). The ImageQuant LAS 4000 digital imaging system (GE Healthcare Europe GmbH, Freiburg, Germany) was used for acquisition of images and Quantity One^®^ v4.6.3 (Bio-Rad Laboratories) for band densitometry. The primary antibodies used for immunodetection were the anti-FADD antibody (FADD (1F7): 05-486; Merck Millipore, Billerica, MA, USA) and anti-DIABLO (Smac (C-10): sc-393118; Santa Cruz Biotechnology, Dallas, TX, USA). The secondary antibody was anti-mouse IgG-HRP (#7074; Cell Signaling Technology, Danvers, MA, USA).

### 4.8. Sample Preparation for LC-MS/MS

Samples were lysed in 5% SDS in 50 mM TEAB supplemented with cOmplete, an EDTA-free Protease Inhibitor Cocktail (Sigma-Aldrich), 1.2 mM Sodium Molybdate, 1 mM Sodium Orthovanadate, 4 mM Sodium Tartrate Dihydrate, 5 mM Glycerophosphate, 20 mM N-ethylmaleimide, and 1 unit/µL Universal Nuclease (Thermo Scientific). Samples were prepared for mass spectrometry using S-Trap micro-columns (Protifi, Farmingdale, NY, USA) according to the manufacturer’s recommended protocol. Proteins were reduced by 20 mM TCEP at 37 °C for 30 min and alkylated by 20 mM N-ethylmaleimide in the dark for 30 min. A ratio of 1:10 wt:wt TPCK-Treated Trypsin (Worthington Biochemical Corp., Lakewood, NJ, USA) was used to digest the samples for 2 h at 47 °C. Eluted peptides were dried down and resuspended in loading buffer (2% acetonitrile, 0.1% trifluoroacetic acid).

### 4.9. Data-Independent Acquisition Mass Spectrometry (DIA-MS)

Peptide samples were injected on a Dionex Ultimate 3000 RSLC (Thermo Fisher Scientific) connected to an Orbitrap FusionTM LumosTM TribridTM mass spectrometer (Thermo Fisher Scientific). Samples were injected on a PepMap 100 C18 LC trap column (300 µm ID × 5 mm, 5 µm, 100 Å) followed by separation on an EASY-Spray column (50 cm × 75 µm ID, PepMap C18, 2 µm, 100 Å) (Thermo Fisher Scientific). Buffer A consisted of water containing 0.1% FA and Buffer B of 80% acetonitrile containing 0.1% FA. Peptides were separated with a liner gradient of 3–35% Buffer B over 115 min followed by a step from 35–90% Buffer B in 10.5 min at 250 nL/min and held at 90% for 4.5 min. The gradient was then decreased to 3% Buffer B in 0.5 min at 250 nL/min for 10 min. Column temperature was controlled at 45 °C. The Orbitrap FusionTM LumosTM TribridTM mass spectrometer was operated in positive ion mode. MS scan spectra were acquired in the range of *m*/*z* 390 to 1010 with a standard automatic gain control (AGC) target and a maximum injection time of 55 ms, at a resolution of 60,000. Targeted MS2 scan spectra were acquired in the range of *m*/*z* 200 to 1600 using 8 *m*/*z* quadrupole isolation windows, AGC of 1000%, at a resolution of 15,000, and higher-energy collision-induced dissociation (HCD) fragmentation was performed in one-step collision energy of 33%. An electrospray voltage was static with a capillary temperature of 275 °C, with an expected LC peak width of 20 s. No sheath and auxiliary gas flow was used.

### 4.10. MS Data Analysis

DIA files were processed on Spectronaut (version 15) performing the directDIATM experimental analysis workflow using default settings with N-ethylmaleimide on cysteine as fixed modification, searched against a SwissProt Homo sapiens database (containing 42,393 database entries with isoforms; downloaded on 14 March 2021). Trypsin specificity was set to two missed cleavages and a protein and PSM false discovery rate of 1%, respectively. Data filtering was set to a Q-value without imputation and normalization set to global normalization. The statistical analysis was done using the R package Limma with a Q-value (adjusted *p*-value) threshold of adjusted *p*-value < 0.05 [64]. The functional enrichment analysis was performed using g:Profiler with a g:SCS multiple testing correction method applying a significance threshold of 0.05 [65]. Protein network visualisation was performed using STRING [66].

### 4.11. Data Availability

The mass spectrometry proteomics data were deposited to the ProteomeXchange Consortium (http://proteomecentral.proteomexchange.org (accessed on 31 October 2022)) via the PRIDE partner repository [67] with the data set identifier PXD037858.

### 4.12. Statistical Analysis

Kaplan–Meier curves with disease-free survival, according to *FADD* expression, were generated by using Graph Pad Prisms v8 (GraphPad Software Inc., La Jolla, CA, USA) with public data available in the Ref. [29]. Best threshold cut-offs were selected automatically by the program. The statistical significance of the distinct experiments was determined after a normality test by using Student’s *t*-test, one-way ANOVA, or the Krustal–Wallis test, using Graph Pad Prisms v8.

## Data Availability

The mass spectrometry proteomics data have been deposited to the ProteomeXchange Consortium (http://proteomecentral.proteomexchange.org (accessed on 31 October 2022)) via the PRIDE partner repository [67] with the data set identifier PXD037858.

## Supplementary Materials

The supporting information can be downloaded at: https://www.mdpi.com/article/10.3390/ijms232315157/s1.
