# Supplementary material for: A Dual Role for FADD in Human Precursor T-Cell Neoplasms
Source: Int J Mol Sci. 2022 Dec 2;23(23):15157. doi: 10.3390/ijms232315157 (PMC9738522; doi:10.3390/ijms232315157)
Supplement: Supplementary file 1 [file ijms-23-15157-s001.zip › Supplementary Figures.pdf]

SUPPLEMENTARY FIGURES

Supplementary Figure S1.

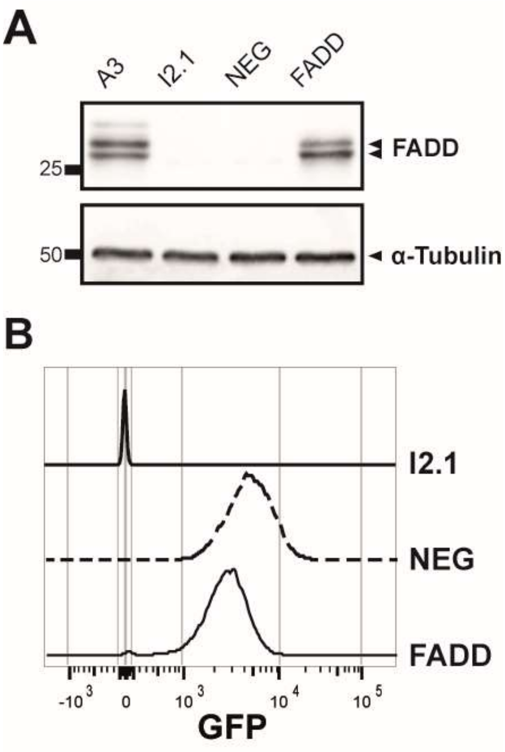

Supplementary Figure S1. FADD expression in the FADD-expressing (FADD) and FADD-deficient (NEG) JURKAT cell lines. A) FADD was detected by western blot in JURKAT clone A3 (A3), JURKAT clone I 2.1 (I2.1) and in the FADD-expressing (FADD) and FADD-deficient (NEG) JURKAT cell lines. NEG, cell line expressing the empty vector; FADD, cell line expressing wild-type FADD. α-Tubulin indicated equivalent loading of samples. Relative mobilities of reference proteins (masses in kiloDaltons) are shown on the left of each blot. **B)** GFP fluorescence obtained by flow cytometry for each stable cell line, using I2.1 as a negative control.

Supplementary Figure S2.

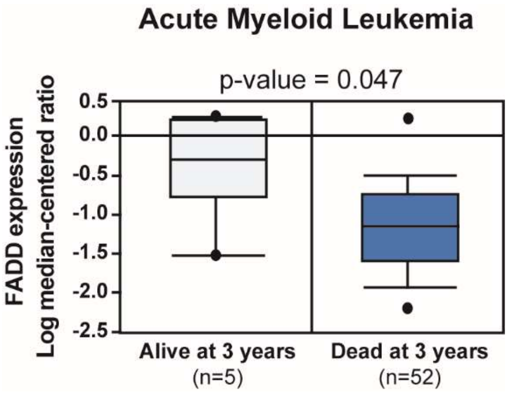

Supplementary Figure S2. FADD expression in AML according to 3-year survival. Box plot illustrating FADD fold expression levels in both alive AML patients at 3-year AML and dead AML patients at 3-years groups. Boxes represent the interquartile range. The lines inside the box represent the median. Whiskers represent minimum and maximum values.

Supplementary Figure S3.

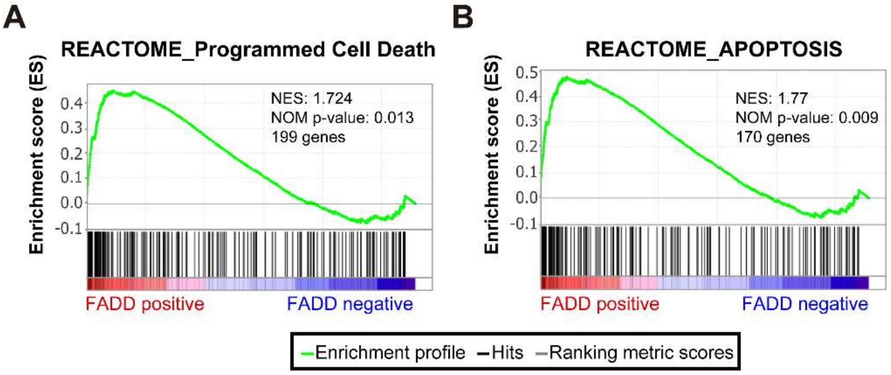

Supplementary Figure S3. Significant association of apoptotic genetic signatures in the FADD-positive phenotype. Gene Set Enrichment Analysis (GSEA) was performed in 264 patients with T-cell lymphoblastic neoplasm, based on their FADD expression levels, which defined FADD-negative and FADD-positive phenotypes. These signatures were significantly enriched in the FADD-positive phenotype. These signatures were selected from the Molecular Signatures Database (MSigDB) and their systematic names are M27436 (Reactome_Programmed Cell Death) and M15303 (Reactome_Apoptosis). The green curve corresponds to the ES (enrichment score) curve, which is the running sum of the weighted enrichment score obtained with the GSEA software. NES, normalized enrichment score; p, nominal p value.
