# Supplementary material for: A Dual Role for FADD in Human Precursor T-Cell Neoplasms
Source: Int J Mol Sci. 2022 Dec 2;23(23):15157. doi: 10.3390/ijms232315157 (PMC9738522; doi:10.3390/ijms232315157)
Supplement: Supplementary file 1 [file ijms-23-15157-s001.zip › Supplementary Table S1.pdf]

**Supplementary Table S1.** List of genes included in the "T-ALL oncogenic signature". Created *ad-hoc* based on previous literature [30].
**SYMBOL**
ABL1
CCND2
DNMT3A
EML1
ETV6
HES1
IL7R
JAK1
JAK2
JAK3
KMT2A
KRAS
LMO2
LYL1
MLLT1
MLLT10
MYB
MYC
NKX3-1
NOTCH1
NRAS
PICALM
PIK3CD
PIK3CG
PTPN11
TAL2
TLX1
TLX3
TRIB2
