# Supplementary material for: A Dual Role for FADD in Human Precursor T-Cell Neoplasms
Source: Int J Mol Sci. 2022 Dec 2;23(23):15157. doi: 10.3390/ijms232315157 (PMC9738522; doi:10.3390/ijms232315157)
Supplement: Supplementary file 1 [file ijms-23-15157-s001.zip › Supplementary Table S2.pdf]

**Supplementary Table S2.** FADD interactors with a significant change in abundance from DIA-MS analysis. The following information is included: Uniprot Ids, protein identifier. Gene names, names of genes this peptide is associated with. Protein name, names of proteins this peptide is associated with. Peptides, the total number of peptide sequences associate with te protein group (i.e. for all the proteins in the group). Log2 Fold Change, Log2 protein fold change. Adj.P-Value, Q-value corrected p-value. Differentially expressed, significant proteins (log2 fold-change ≤ +/-0.6; adjusted p-value <0.05). Log2 LFQ intensity, Log2 LFQ reporter intensities. Summed up eXtracted Ion Current (XIC) of all isotopic clusters associated with the identified AA sequence.

| Uniprot Ids | Gene names | Protein name | Peptides | Log2 Fold Change | adj.P-Value | Log2 LFQ intensity FADD WT Rep_1 | Log2 LFQ intensity FADD WT Rep_2 | Log2 LFQ intensity FADD WT Rep_3 | Log2 LFQ intensity FADD WT Rep_4 | Log2 LFQ intensity FADD KO Rep_1 | Log2 LFQ intensity FADD KO Rep_2 | Log2 LFQ intensity FADD KO Rep_3 | Log2 LFQ intensity FADD KO Rep_4 |
|---|---|---|---|---|---|---|---|---|---|---|---|---|---|
| Q9UKZ1 | CNOT11 | CCR4-NOT transcription complex subunit | 2 | 5,81 | 3,19E-03 | 15,67 | 15,48 | 12,39 | 12,98 | 14,10 | 13,92 | 2,77 | 2,50 |
| Q9BX67 | JAM3 | Junctional adhesion molecule C | 5 | 5,68 | 5,70E-03 | 15,49 | 16,31 | 12,09 | 14,20 | 14,96 | NA | 2,72 | NA |
| O75531 | BANF1 | Barrier-to-autointegration factor | 3 | 5,02 | 9,71E-03 | 9,29 | 11,90 | NA | 9,56 | -0,10 | 10,56 | NA | NA |
| Q9Y5Z4 | HEBP2 | Heme-binding protein 2 | 10 | 4,52 | 1,49E-02 | 12,88 | 16,96 | NA | NA | 12,15 | 3,87 | 14,06 | 11,50 |
| P34059 | GALNS | N-acetylgalactosamine-6-sulfatase | 3 | 4,31 | 2,87E-02 | 13,21 | 14,48 | NA | 12,02 | 11,79 | 13,86 | NA | 1,13 |
| Q8TDQ7;Q8TDQ7-3 | GNPDA2 | Glucosamine-6-phosphate isomerase 2;I | 3 | 4,21 | 1,88E-02 | 12,89 | 13,76 | 11,42 | 13,86 | 11,84 | 13,38 | 1,10 | NA |
| Q96K17 | BTF3L4 | Transcription factor BTF3 homolog 4 | 3 | 3,67 | 3,10E-02 | 11,21 | 14,16 | 10,18 | 12,82 | NA | 13,25 | 3,59 | NA |
| Q14684;Q14684-2 | RRP1B | Ribosomal RNA processing protein 1 hor | 4 | 3,63 | 2,90E-03 | 15,63 | 15,76 | NA | 18,55 | 14,09 | NA | NA | 11,93 |
| P20290-2 | BTF3 | Isoform 2 of Transcription factor BTF3 | 11 | 3,51 | 6,68E-04 | 18,48 | 17,71 | 16,01 | 14,78 | 14,22 | 12,10 | 15,20 | 11,41 |
| Q6UWI4 | SHISA2 | Protein shisa-2 homolog | 2 | 3,25 | 2,03E-02 | NA | 16,59 | NA | NA | 14,72 | 12,95 | 12,34 | NA |
| P17174 | GOT1 | Aspartate aminotransferase, cytoplasmic | 21 | 3,19 | 5,59E-03 | 14,73 | 14,61 | 12,63 | 13,81 | 6,29 | 13,00 | 12,89 | 10,87 |
| Q9NRR3 | CDC42SE2 | CDC42 small effector protein 2 | 2 | 3,19 | 7,59E-03 | 14,54 | 15,72 | NA | NA | 10,85 | 14,10 | NA | 10,87 |
| P98155;P98155-2 | VLDLR | Very low-density lipoprotein receptor;Is | 15 | 3,18 | 7,44E-03 | 13,64 | 12,53 | 10,88 | 12,26 | 4,62 | 12,36 | 10,56 | 9,07 |
| Q9P0W2;Q9P0W2-2;Q9P0W2-3 | HMG20B | SWI/SNF-related matrix-associated actin | 3 | 3,15 | 8,91E-04 | 15,34 | 15,88 | NA | 14,34 | 10,83 | 13,54 | 11,67 | 12,10 |
| Q5JS54;Q5JS54-2 | PSMG4 | Proteasome assembly chaperone 4;Isofo | 2 | 3,09 | 2,71E-02 | NA | 14,15 | NA | NA | 9,83 | 12,39 | 10,94 | NA |
| Q6PEY2 | TUBA3E | Tubulin alpha-3E chain | 3 | 3,09 | 1,46E-02 | 18,68 | 18,63 | 21,63 | 17,70 | 17,99 | 18,04 | 17,52 | 10,73 |
| O95218;O95218-2 | ZRANB2 | Zinc finger Ran-binding domain-containi | 2 | 2,99 | 1,11E-02 | 14,60 | 13,07 | 11,10 | 13,08 | 5,33 | 11,89 | 10,50 | 12,18 |
| Q14197 | MRPL58 | Peptidyl-tRNA hydrolase ICT1, mitochon | 6 | 2,95 | 1,65E-02 | 17,53 | 19,01 | 16,29 | 15,77 | 16,23 | 18,07 | 10,83 | 11,67 |
| Q13158 | FADD | FAS-associated death domain protein | 6 | 2,93 | 1,37E-03 | 18,34 | 17,08 | 15,72 | 15,54 | 14,62 | 13,13 | 14,93 | 12,25 |
| Q5VZL5;Q5VZL5-2;Q5VZL5-3;Q5VZL5-4 | ZMYM4 | Zinc finger MYM-type protein 4;Isoform | 2 | 2,92 | 1,34E-03 | 14,73 | 13,90 | 13,97 | 14,95 | 13,16 | 12,31 | 11,14 | 9,27 |
| Q5VTL8;Q5VTL8-2 | PRPF38B | Pre-mRNA-splicing factor 38B;Isoform 2 | 3 | 2,89 | 2,30E-03 | 13,05 | NA | 12,69 | 13,60 | 9,66 | 10,33 | NA | 10,70 |
| P46109 | CRKL | Crk-like protein | 12 | 2,88 | 5,97E-03 | 14,82 | 15,60 | 12,49 | NA | 10,13 | 12,28 | 11,87 | NA |
| P17050 | NAGA | Alpha-N-acetylgalactosaminidase | 7 | 2,86 | 4,54E-02 | 14,22 | 17,89 | NA | 13,34 | NA | 14,87 | 9,72 | NA |
| Q9Y3T9 | NOC2L | Nucleolar complex protein 2 homolog | 2 | 2,85 | 4,46E-02 | 16,05 | 19,08 | NA | 17,58 | 14,72 | NA | NA | NA |
| Q8IYB3;Q8IYB3-2 | SRRM1 | Serine/arginine repetitive matrix protein | 6 | 2,84 | 9,07E-03 | 13,23 | 14,47 | NA | 13,61 | 11,68 | 8,40 | NA | 12,71 |
| Q9UHG3 | PCYOX1 | Prenylcysteine oxidase 1 | 17 | 2,82 | 1,71E-03 | 14,19 | 14,04 | 16,61 | 12,92 | 11,01 | 12,65 | 11,64 | 11,17 |
| Q9H0K6 | PUS7L | Pseudouridylate synthase 7 homolog-like | 7 | 2,80 | 2,28E-02 | 15,38 | 16,59 | 12,88 | 14,36 | 14,07 | 15,25 | 7,38 | 11,29 |
| Q9BRP8;Q9BRP8-2 | PYM1 | Partner of Y14 and mago;Isoform 2 of Pa | 4 | 2,71 | 1,47E-02 | 14,82 | 14,67 | 12,62 | 15,13 | 10,08 | 14,93 | 9,81 | NA |
| Q6BDS2 | UHRF1BP1 | UHRF1-binding protein 1 | 2 | 2,70 | 6,64E-03 | 14,01 | 12,30 | NA | 13,40 | 9,95 | 12,02 | NA | 9,65 |
| O14646;O14646-2 | CHD1 | Chromodomain-helicase-DNA-binding pr | 5 | 2,66 | 7,70E-03 | 15,13 | NA | 15,94 | 13,81 | 13,44 | 12,30 | 9,90 | 13,58 |
| Q9BQ39 | DDX50 | ATP-dependent RNA helicase DDX50 | 5 | 2,64 | 6,36E-03 | 16,11 | 15,70 | NA | 14,42 | 13,82 | NA | 12,05 | 12,44 |
| O95159 | ZFPL1 | Zinc finger protein-like 1 | 2 | 2,61 | 3,30E-03 | 15,18 | 14,54 | 14,82 | 13,83 | 12,88 | 13,67 | 11,38 | 9,99 |
| Q9Y2A7;Q9Y2A7-2 | NCKAP1 | Nck-associated protein 1;Isoform 2 of Nc | 3 | 2,60 | 1,18E-02 | 14,61 | 14,99 | NA | 13,92 | 12,54 | 14,50 | 11,10 | 9,48 |
| Q9Y2G8 | DNAJC16 | DnaJ homolog subfamily C member 16 | 2 | 2,60 | 5,85E-03 | 13,72 | 13,57 | NA | 13,32 | 11,64 | 11,26 | NA | 9,90 |
| Q96AE4-2 | FUBP1 | Isoform 2 of Far upstream element-bind | 2 | 2,55 | 2,26E-02 | 13,82 | 17,15 | NA | 13,23 | 10,18 | 15,13 | 11,58 | 11,82 |
| P30825 | SLC7A1 | High affinity cationic amino acid transpo | 4 | 2,54 | 1,46E-02 | 13,37 | 14,68 | 13,45 | 13,81 | NA | 14,02 | 11,10 | 8,74 |
| P14174 | MIF | Macrophage migration inhibitory factor | 2 | 2,54 | 6,61E-03 | 16,65 | 18,00 | 17,24 | 16,31 | 11,87 | 16,55 | 14,31 | 15,31 |
| Q5JU69 | TOR2A | Torsin-2A | 2 | 2,54 | 2,46E-02 | 16,50 | 17,27 | NA | 15,16 | 15,50 | 16,43 | 10,87 | 12,30 |
| Q6JBY9 | RCSD1 | CapZ-interacting protein | 10 | 2,52 | 8,07E-03 | 14,37 | 17,76 | 16,31 | 16,65 | 12,95 | 13,51 | 16,26 | 12,31 |
| Q9ULP9;Q9ULP9-2 | TBC1D24 | TBC1 domain family member 24;Isoform | 4 | 2,49 | 3,40E-02 | 13,62 | 13,79 | 11,14 | 14,78 | 11,90 | 12,94 | 6,01 | 12,52 |
| Q969F9;Q969F9-2 | HPS3 | Hermansky-Pudlak syndrome 3 protein;I | 3 | 2,49 | 9,90E-03 | 15,08 | 14,20 | NA | 13,63 | 12,46 | 12,47 | NA | 10,52 |
| A6NDU8 | RIMOC1 | RAB7A-interacting MON1-CCZ1 complex | 3 | 2,41 | 2,89E-02 | 14,24 | 15,57 | NA | 13,07 | 11,19 | 14,44 | NA | 10,01 |
| Q567V2;Q567V2-2 | MPV17L2 | Mpv17-like protein 2;Isoform 2 of Mpv1 | 2 | 2,41 | 2,14E-02 | 16,07 | 16,47 | NA | 15,44 | 14,28 | 15,28 | NA | 11,20 |
| Q9C0D9 | SELENOI | Ethanolaminephosphotransferase 1 | 2 | 2,39 | 1,44E-02 | 14,72 | 15,99 | NA | 14,65 | 13,33 | 13,66 | NA | 11,21 |
| P21675;P21675-12;P21675-2;P21675-3;P21675-4;P21675-5;P21675-6;P21675-7;P21675-8;P21675-9;Q8IZX4 | TAF1;TAF1;TAF1; | Transcription initiation factor TFIID subu | 5 | 2,37 | 4,27E-02 | 14,57 | 12,60 | NA | 13,00 | 13,96 | 10,93 | NA | 8,17 |
| P50897 | PPT1 | Palmitoyl-protein thioesterase 1 | 8 | 2,36 | 1,76E-02 | 15,91 | 15,30 | 13,45 | 14,38 | 13,39 | 13,89 | 13,42 | 8,89 |
| Q6NUM9;Q6NUM9-2 | RETSAT | All-trans-retinol 13,14-reductase;Isoform | 4 | 2,35 | 1,49E-02 | 14,84 | 15,27 | 12,85 | 14,41 | 8,98 | 13,44 | 11,99 | 13,57 |
| Q9Y375 | NDUFAF1 | Complex I intermediate-associated prote | 4 | 2,28 | 2,53E-02 | 15,06 | 15,97 | NA | 13,68 | 13,88 | 14,42 | 12,14 | 10,04 |
| Q8N8N7 | PTGR2 | Prostaglandin reductase 2 | 3 | 2,24 | 1,75E-02 | 13,84 | 14,43 | 14,51 | 11,60 | 10,43 | 12,65 | NA | 10,99 |
| Q9Y3E1 | HDGFL3 | Hepatoma-derived growth factor-related | 4 | 2,22 | 4,88E-02 | 15,58 | 13,02 | NA | 12,17 | 11,11 | 11,63 | NA | NA |
| Q9Y2R4 | DDX52 | Probable ATP-dependent RNA helicase D | 2 | 2,21 | 3,20E-02 | 15,68 | 12,93 | NA | 14,33 | 13,94 | 11,17 | NA | 11,22 |
| P46976;P46976-2 | GYG1 | Glycogenin-1;Isoform GN-1 of Glycogeni | 7 | 2,20 | 2,55E-02 | 14,00 | 14,44 | NA | 14,89 | 12,65 | 13,66 | 10,44 | NA |
| Q9HAU0;Q9HAU0-2;Q9HAU0-3;Q9HAU0-4;Q9HAU0-5;Q9HAU0-6 | PLEKHA5 | Pleckstrin homology domain-containing | 2 | 2,18 | 1,70E-02 | 15,47 | 15,75 | 14,22 | 15,11 | 11,57 | 14,74 | 10,97 | 14,55 |
| O96000 | NDUFB10 | NADH dehydrogenase [ubiquinone] 1 be | 2 | 2,17 | 3,21E-02 | 15,92 | 15,91 | 14,71 | NA | 14,76 | 15,20 | 10,59 | 12,80 |
| Q8NG31;Q8NG31-2 | KNL1 | Kinetochore scaffold 1;Isoform 2 of Kine | 3 | 2,17 | 3,16E-02 | 14,01 | 13,35 | NA | 12,56 | 12,22 | 12,15 | NA | 9,06 |
| P27144 | AK4 | Adenylate kinase 4, mitochondrial | 3 | 2,16 | 2,23E-02 | 16,74 | 16,43 | 14,27 | 14,24 | 14,58 | 14,28 | 13,68 | 10,49 |
| Q8N9N8 | EIF1AD | Probable RNA-binding protein EIF1AD | 3 | 2,16 | 4,94E-02 | 13,45 | 14,45 | NA | NA | 11,26 | 13,49 | 10,64 | NA |
| Q9C0D5;Q9C0D5-2 | TANC1 | Protein TANC1;Isoform 2 of Protein TAN | 3 | 2,16 | 8,11E-03 | 15,92 | 15,72 | 14,39 | 14,62 | 14,23 | 12,93 | 11,83 | 13,02 |
| E9PQ53;O95298;O95298-2;O95298-3 | NDUFC2-KCTD14 | NADH dehydrogenase [ubiquinone] 1 su | 2 | 2,16 | 3,71E-02 | 17,28 | 17,80 | 15,83 | NA | 15,47 | 16,56 | 15,55 | 11,67 |
| P05387 | RPLP2 | 60S acidic ribosomal protein P2 | 8 | 2,14 | 2,13E-02 | 15,27 | 15,26 | 15,78 | 13,94 | 11,18 | 14,57 | NA | 13,02 |
| O15116 | LSM1 | U6 snRNA-associated Sm-like protein LSr | 2 | 2,14 | 4,74E-02 | 13,20 | 14,20 | NA | 13,76 | NA | 12,90 | NA | 10,26 |
| Q92854 | SEMA4D | Semaphorin-4D | 9 | 2,14 | 2,29E-02 | 11,48 | 12,53 | NA | 10,78 | 10,13 | 9,75 | NA | 8,50 |
| Q8N8A6 | DDX51 | ATP-dependent RNA helicase DDX51 | 8 | 2,13 | 3,00E-02 | 16,43 | 15,08 | NA | 14,14 | 14,59 | 13,65 | 10,57 | 13,54 |
| Q71F23;Q71F23-3 | CENPU | Centromere protein U;Isoform 3 of Cent | 2 | 2,12 | 2,40E-02 | 13,94 | 13,22 | NA | 12,17 | 12,95 | 10,12 | 9,52 | 11,37 |
| Q92504 | SLC39A7 | Zinc transporter SLC39A7 | 2 | 2,11 | 2,43E-02 | 12,15 | NA | 12,81 | 13,86 | 9,80 | NA | 11,31 | 11,37 |
| Q86VQ1 | GLCCI1 | Glucocorticoid-induced transcript 1 prot | 2 | 2,11 | 3,18E-02 | 15,27 | 17,19 | 13,66 | 15,26 | 13,90 | 15,69 | 10,61 | 12,73 |

| | | | | | | | | | | | | | |
|---|---|---|---|---|---|---|---|---|---|---|---|---|---|
| Q9NRA8;Q9NRA8-3 | EIF4ENIF1 | Eukaryotic translation initiation factor 4E | 3 | 2,11 | 1,32E-02 | 15,41 | 15,40 | 15,61 | 14,72 | 13,63 | 14,38 | 13,83 | 10,87 |
| Q8IWA4 | MFN1 | Mitofusin-1 | 2 | 2,10 | 4,19E-02 | 15,41 | 16,02 | NA | 14,05 | 13,41 | 14,79 | NA | 10,97 |
| P11279 | LAMP1 | Lysosome-associated membrane glycopr | 7 | 2,09 | 2,26E-02 | 14,10 | 14,39 | 15,85 | 13,37 | 11,85 | 13,86 | 11,29 | NA |
| P29374;P29374-2;P29374-3 | ARID4A | AT-rich interactive domain-containing pr | 2 | 2,08 | 4,83E-02 | 15,47 | 15,58 | 12,12 | 12,97 | 13,06 | 13,26 | NA | 9,54 |
| Q8WXA9-2 | SREK1 | Isoform 2 of Splicing regulatory glutamin | 7 | 2,06 | 1,96E-02 | 15,19 | 14,18 | 13,40 | 15,09 | 12,86 | 11,63 | 10,57 | 14,54 |
| Q86T03;Q86T03-2 | PIP4P1 | Type 1 phosphatidylinositol 4,5-bisphosp | 2 | 2,04 | 3,75E-02 | 14,29 | 14,90 | 11,41 | 14,63 | 12,79 | 13,19 | 8,78 | 12,31 |
| Q7L8L6 | FASTKD5 | FAST kinase domain-containing protein 5 | 2 | 2,04 | 3,94E-02 | 16,31 | 15,22 | 13,03 | 15,07 | 14,33 | 15,19 | 10,42 | 11,52 |
| Q9Y3R5;Q9Y3R5-2 | DOP1B | Protein dopey-2;Isoform 2 of Protein do | 3 | 2,04 | 4,84E-02 | 14,10 | 14,16 | 14,10 | 13,56 | 12,98 | 13,60 | 7,63 | 13,55 |
| Q8N5I9 | NOPCHAP1 | NOP protein chaperone 1 | 4 | 2,04 | 3,25E-02 | 14,00 | 15,78 | 13,05 | 14,06 | 12,70 | 14,39 | 12,33 | 9,32 |
| Q96FX7 | TRMT61A | tRNA (adenine(58)-N(1))-methyltransfer | 4 | 2,03 | 2,21E-02 | 14,93 | 15,05 | NA | 15,28 | 13,76 | 14,06 | 11,24 | 13,17 |
| Q96NW4 | ANKRD27 | Ankyrin repeat domain-containing prote | 2 | 2,01 | 1,51E-02 | 16,06 | 15,73 | 13,35 | 15,25 | 13,46 | 13,38 | 11,92 | 13,58 |
| Q92771;Q96FC9;Q96FC9-2;Q96FC9-3;Q96FC9-4 | DDX12P;DDX11;D | Putative ATP-dependent RNA helicase D | 3 | 1,98 | 3,24E-02 | 15,51 | 15,56 | NA | 14,26 | 14,47 | 14,32 | 11,36 | 12,36 |
| P53367;P53367-2 | ARFIP1 | Arfaptin-1;Isoform A of Arfaptin-1 | 7 | 1,98 | 1,88E-02 | 13,46 | 14,00 | 11,30 | 13,18 | 11,07 | 12,52 | 10,31 | 10,11 |
| P0DP23;P0DP24;P0DP25 | CALM1;CALM2;C | Calmodulin-1;Calmodulin-2;Calmodulin-3 | 5 | 1,94 | 3,91E-02 | 11,93 | 14,00 | NA | 10,02 | 10,42 | 10,69 | 9,45 | 9,64 |
| Q9NRX4 | PHPT1 | 14 kDa phosphohistidine phosphatase | 4 | 1,93 | 4,76E-02 | 15,40 | 16,32 | 12,38 | 14,62 | 13,35 | 15,12 | 10,65 | 11,88 |
| Q02338 | BDH1 | D-beta-hydroxybutyrate dehydrogenase, | 2 | 1,91 | 2,55E-02 | 16,64 | 16,76 | 14,67 | 15,38 | 14,52 | 15,67 | 12,89 | 12,73 |
| Q8IWV8;Q8IWV8-4 | UBR2 | E3 ubiquitin-protein ligase UBR2;Isoform | 7 | 1,88 | 4,03E-02 | 15,04 | 14,08 | 15,30 | 13,76 | 13,88 | 14,32 | 9,73 | 12,74 |
| P43307 | SSR1 | Translocon-associated protein subunit al | 3 | 1,87 | 4,89E-02 | 18,21 | 17,78 | 15,52 | 15,63 | 16,64 | 16,37 | 12,43 | 14,20 |
| Q9NQW6;Q9NQW6-2 | ANLN | Anillin;Isoform 2 of Anillin | 3 | 1,87 | 3,70E-02 | 13,22 | 13,50 | 11,03 | 13,78 | 11,03 | 11,78 | NA | 10,24 |
| Q9BYV8;Q9BYV8-2 | CEP41 | Centrosomal protein of 41 kDa;Isoform 2 | 2 | 1,86 | 1,63E-02 | 14,45 | 15,46 | 13,91 | 14,06 | 12,39 | 13,27 | 12,89 | 11,90 |
| P84090 | ERH | Enhancer of rudimentary homolog | 4 | 1,85 | 3,67E-02 | 14,12 | 14,44 | 11,49 | 13,66 | 11,40 | 13,63 | 10,19 | 11,08 |
| Q2VPB7 | AP5B1 | AP-5 complex subunit beta-1 | 4 | 1,84 | 2,48E-02 | 15,80 | 15,51 | 13,06 | 14,27 | 12,82 | 13,62 | 12,93 | 11,90 |
| P04792 | HSPB1 | Heat shock protein beta-1 | 3 | 1,84 | 2,28E-02 | 13,66 | 15,23 | 14,48 | 16,26 | 13,16 | 13,58 | 13,52 | 12,00 |
| Q7Z6L1;Q7Z6L1-2 | TECPR1 | Tectonin beta-propeller repeat-containin | 3 | 1,83 | 4,92E-02 | 15,68 | 15,95 | NA | 13,80 | 14,64 | 14,35 | 11,79 | 12,50 |
| O60508 | CDC40 | Pre-mRNA-processing factor 17 | 6 | 1,81 | 3,37E-02 | 16,03 | 15,22 | NA | 15,75 | 14,05 | 14,00 | 12,53 | 14,84 |
| O43426;O43426-2;O43426-4 | SYNJ1 | Synaptojanin-1;Isoform 2 of Synaptojani | 3 | 1,80 | 1,80E-02 | 14,35 | 14,99 | 13,49 | 14,64 | 12,43 | 13,07 | 12,45 | 12,34 |
| P25445;P25445-6 | FAS | Tumor necrosis factor receptor superfam | 2 | 1,77 | 3,02E-02 | 13,53 | 13,63 | 13,15 | 13,18 | 11,08 | NA | 11,61 | 12,12 |
| P30040 | ERP29 | Endoplasmic reticulum resident protein | 11 | 1,77 | 3,22E-02 | 13,86 | 13,56 | 13,24 | 13,99 | 12,01 | 12,53 | 11,13 | NA |
| Q9BUL8 | PDCD10 | Programmed cell death protein 10 | 7 | 1,74 | 4,83E-02 | 13,92 | 15,32 | 13,27 | NA | 11,50 | 13,54 | 13,10 | 11,57 |
| Q99638 | RAD9A | Cell cycle checkpoint control protein RAD | 2 | 1,74 | 4,89E-02 | 16,29 | 16,83 | NA | 16,58 | 15,14 | 15,54 | 12,88 | 15,74 |
| Q9Y3E0 | GOLT1B | Vesicle transport protein GOT1B | 3 | 1,73 | 4,59E-02 | 16,67 | 17,04 | NA | 15,86 | 15,54 | 15,92 | 13,62 | 14,09 |
| P04049 | RAF1 | RAF proto-oncogene serine/threonine-p | 6 | 1,73 | 4,40E-02 | 15,89 | 16,17 | 14,34 | 13,76 | 14,13 | 14,75 | 12,67 | 11,70 |
| Q9Y4D8;Q9Y4D8-4 | HECTD4 | Probable E3 ubiquitin-protein ligase HEC | 4 | 1,69 | 3,30E-02 | 14,28 | 14,58 | 13,44 | 14,17 | 13,56 | 13,23 | 11,21 | 11,69 |
| Q7Z6M1 | RABEPK | Rab9 effector protein with kelch motifs | 4 | 1,69 | 3,39E-02 | 15,25 | 16,28 | 14,61 | 15,27 | 14,12 | 14,95 | 12,95 | 12,61 |
| Q9NR28;Q9NR28-2;Q9NR28-3 | DIABLO | Diablo IAP-binding mitochondrial protein | 2 | 1,68 | 3,80E-02 | 18,13 | 18,80 | 17,98 | 17,85 | 16,34 | 16,51 | 16,67 | NA |
| O75648;O75648-2;O75648-3;O75648-4 | TRMU | Mitochondrial tRNA-specific 2-thiouridyl | 2 | 1,68 | 3,38E-02 | 15,25 | 15,24 | 14,84 | 14,35 | 12,38 | 14,29 | 14,16 | 12,15 |
| Q14554 | PDIA5 | Protein disulfide-isomerase A5 | 11 | 1,68 | 4,61E-02 | 16,70 | 15,81 | 14,30 | 15,42 | 15,30 | 14,59 | 12,22 | 13,41 |
| Q12797;Q12797-10 | ASPH | Aspartyl/asparaginyl beta-hydroxylase;Is | 4 | 1,68 | 4,22E-02 | 13,94 | 14,76 | 11,59 | 13,96 | 12,57 | 12,29 | 10,91 | 11,77 |
| Q96MH6 | TMEM68 | Transmembrane protein 68 | 4 | 1,66 | 3,63E-02 | 14,27 | 15,74 | 13,69 | 14,69 | 12,65 | 14,17 | 11,99 | 12,92 |
| P49336;P49336-2;Q9BWU1;Q9BWU1-2 | CDK8;CDK8;CDK1 | Cyclin-dependent kinase 8;Isoform 2 of C | 2 | 1,64 | 3,19E-02 | 16,66 | 16,93 | 15,71 | 16,04 | 15,27 | 15,22 | 13,75 | 14,55 |
| P78563;P78563-3;P78563-5 | ADARB1 | Double-stranded RNA-specific editase 1; | 2 | 1,64 | 3,70E-02 | 14,47 | 14,30 | 13,39 | 14,99 | 13,86 | 12,99 | 11,85 | 11,89 |
| Q8NBJ7 | SUMF2 | Inactive C-alpha-formylglycine-generatin | 10 | 1,64 | 4,35E-02 | 13,49 | 14,36 | 12,50 | 13,40 | 10,86 | 13,45 | 11,97 | 10,92 |
| Q9Y624 | F11R | Junctional adhesion molecule A | 5 | 1,63 | 4,61E-02 | 13,22 | 12,71 | 12,01 | 13,45 | 11,15 | 11,22 | NA | 11,26 |
| Q15043;Q15043-2;Q15043-3 | SLC39A14 | Metal cation symporter ZIP14;Isoform 3 | 2 | 1,63 | 4,96E-02 | 14,45 | 15,66 | 13,59 | 14,34 | 12,19 | 14,87 | 11,90 | 12,57 |
| P25208 | NFYB | Nuclear transcription factor Y subunit be | 2 | 1,62 | 3,28E-02 | 13,86 | 13,02 | 12,89 | 12,90 | 11,56 | 12,56 | 11,11 | 10,95 |
| Q9HBK9 | AS3MT | Arsenite methyltransferase | 3 | 1,58 | 4,63E-02 | 14,76 | 15,59 | 13,70 | 14,72 | 14,19 | 13,76 | 12,08 | 12,42 |
| O15173;O15173-2 | PGRMC2 | Membrane-associated progesterone rec | 7 | 1,57 | 4,42E-02 | 14,03 | 14,30 | 12,56 | 12,31 | 11,36 | 12,41 | 11,43 | 11,74 |
| P49366 | DHPS | Deoxyhypusine synthase | 14 | 1,56 | 4,35E-02 | 15,00 | 15,02 | 13,89 | 14,18 | 12,91 | 14,10 | 12,91 | 11,93 |
| P51970 | NDUFA8 | NADH dehydrogenase [ubiquinone] 1 alp | 5 | 1,55 | 4,14E-02 | 13,92 | 14,50 | 13,15 | 13,17 | 12,29 | 11,92 | 12,91 | 11,40 |
| Q8WWV3 | RTN4IP1 | Reticulon-4-interacting protein 1, mitoch | 6 | 1,52 | 4,96E-02 | 12,89 | 13,69 | 11,43 | 12,32 | 10,53 | 11,61 | 11,30 | 10,83 |
| Q9H2D6;Q9H2D6-2;Q9H2D6-3;Q9H2D6-5;Q9H2D6-7 | TRIOBP | TRIO and F-actin-binding protein;Isoform | 4 | 1,44 | 4,93E-02 | 16,97 | 16,74 | 16,35 | 16,28 | 15,00 | 14,92 | 15,61 | 15,06 |
